# Ketamine as Monotherapy in Difficult Airways Is Not Ready for Prime Time

**DOI:** 10.5811/westjem.2019.8.43881

**Published:** 2019-10-17

**Authors:** Brian E. Driver, Robert F. Reardon, Jarrod Mosier

**Affiliations:** * Hennepin County Medical Center, Department of Emergency Medicine, Minneapolis, Minnesota; † University of Arizona, Department of Emergency Medicine, Section of Pulmonary, Critical Care, Allergy, and Sleep, Department of Medicine, Tucson, Arizona

To the Editor:

We appreciate the discussion outlined by Merelman et al. regarding the important role ketamine has in emergency airway management, 1 and agree with the sentiment that ketamine may be preferable to other agents in many different clinical scenarios. Based on our experience teaching and discussing emergency airway management with national experts, however, we believe a few points are more nuanced and warrant further discussion.

For patients with predicted intubation difficulty, the authors advocate sedation with ketamine and the use of a standard laryngoscope. While this technique may be appropriate in certain clinical scenarios, there is a dearth of evidence demonstrating its success or safety and we recommend further study before it is widely implemented. Intubation with ketamine alone, in the references cited, was successful in 21/31 (68%) cases. 2,3 Fiberoptic intubation success with ketamine monotherapy has also had low success rates. 4 Ketamine may dissociate the cortex from brainstem functions, but because brainstem reflexes remain intact, vomiting can still occur when the upper airway structures are stimulated. Emesis occurs in approximately 5–15% of ketamine administrations in adults, 5 which often leads to aspiration–the largest contributor to morbidity in airway management globally. Ideally, patients thought to be too difficult for neuromuscular blockade are managed with meticulous topical anesthesia and as little parenteral sedation or anxiolysis as feasible; sedation without dissociation or obtundation allows the patient to follow commands, which is advantageous during endoscopic intubation.

Although standard laryngoscopy is the most common emergency intubation technique, we strongly believe that flexible endoscopic intubation is an important skill within the procedural capability of emergency physicians. This has long been the gold standard method for patients deemed too risky for neuromuscular blockade. While video laryngoscopes have largely replaced direct laryngoscopy, the utilization of flexible endoscopy has remained fairly constant. 6 Historically, the expense of flexible fiberoptic scopes and endoscopes hindered widespread access to these important devices; for this reason, many physicians have not received adequate training or ongoing practice, especially in departments that infrequently perform intubation. The advent of disposable endoscopes, now produced by multiple companies, should improve accessibility and affordability. Like any procedure, continual practice with a flexible endoscope is essential. This can be accomplished in many ways that should be feasible by all physicians. In our department we have practiced nasal intubation on each other, which has honed our topical anesthesia skills. Endoscopic evaluation of ED patients with severe sore throats, foreign body sensation, new hoarseness, and other conditions provides practice with endoscope controls; manikin-based practice is another option.

Ketamine, while uncommonly causing overt respiratory depression or apnea, frequently causes subclinical respiratory depression. 7,8 This is inconsequential in patients with normal respiratory effort (eg, procedural sedation of healthy patients), but it is important to consider when caring for critically ill patients. In our experience, when ketamine is administered to patients with high minute ventilation (eg, severe agitation and excited delirium, diabetic ketoacidosis, acute respiratory distress syndrome), they continue to breathe but with a significantly lower minute ventilation that sometimes does not meet their metabolic needs. We believe that patients with high respiratory effort who are deemed too risky for neuromuscular blockade should be managed either with a completely awake approach (ie, no slowing of respiration), or with rapid sequence intubation, which maximizes the chance of first-pass success and allows placement of a first-line backup device (eg, intubating laryngeal mask airway) should the first attempt fail. It may be preferable to cause apnea with neuromuscular blockade rather than risk a longer ketamine-facilitated intubation attempt with relative hypoventilation. The worst possible circumstance when managing these patients is to have a patient who is not breathing adequately and also not relaxed enough to facilitate tube passage or allow placement of a modern extraglottic device.

Ketamine is an old drug that remains valuable in all phases of airway management. Before widespread use as a monotherapy for patients with difficult airways, however, it seems prudent to gather additional data to determine its success and safety profile relative to other approaches.
